# Combined metabolic and enzymatic engineering for *de novo* biosynthesis of δ-tocotrienol in *Yarrowia lipolytica*

**DOI:** 10.1016/j.synbio.2025.02.011

**Published:** 2025-02-20

**Authors:** Jinbo Xiang, Mengsu Liu, Xinglong Wang, Mingyu Yue, Zhijie Qin, Jingwen Zhou

**Affiliations:** a Engineering Research Center of Ministry of Education on Food Synthetic Biotechnology, School of Biotechnology, Jiangnan University, 1800 Lihu Road, Wuxi, Jiangsu, 214122, China; b Science Center for Future Foods, Jiangnan University, 1800 Lihu Rd, Wuxi, Jiangsu 214122, China; c Jiangsu Province Engineering Research Center of Food Synthetic Biotechnology, Jiangnan University, 1800 Lihu Road, Wuxi, Jiangsu, 214122, China

**Keywords:** δ-tocotrienol, *Yarrowia lipolytica*, Metabolic engineering, enzyme engineering, Fed-batch fermentation

## Abstract

δ-Tocotrienol, an isomer of vitamin E with anti-inflammatory, neuroprotective and anti-coronary arteriosclerosis properties, is widely used in health care, medicine and other fields. Microbial synthesis of δ-tocotrienol offers significant advantages over plant extraction and chemical synthesis methods, including increased efficiency, cost-effectiveness and environmental sustainability. However, limited precursor availability and low catalytic efficiency of key enzymes remain major bottlenecks in the biosynthesis of δ-tocotrienol. In this study, we assembled the complete δ-tocotrienol biosynthetic pathway in *Yarrowia lipolytica* and enhanced the precursor supply, resulting in a titre of 102.8 mg/L. The catalytic efficiency of the rate-limiting steps in the pathway was then enhanced through various strategies, including fusion expression of key enzymes homogentisate phytyltransferaseand and tocopherol cyclase, semi-rational design of SyHPT and multi-copy integration of pathway genes. The final a δ-tocotrienol titre in a 5 L bioreactor was 466.8 mg/L following fed-batchfermentation. This study represents the first successful *de novo* biosynthesis of δ-tocotrienol in *Y. lipolytica*, providing valuable insights into the synthesis of vitamin E-related compounds.

## Graphical abstract

**Figure:**
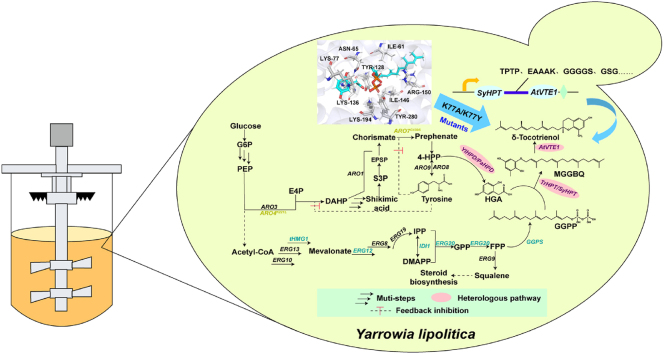

## 1 Introduction

Vitamin E, an essential nutrient for maintaining normal metabolism and bodily functions, primarily comprises tocopherols and tocotrienols, each further divided into α, β, γ and δ [1] subtypes. Among these eight components, δ-tocotrienol stands out due to its potent anti-cancer, anti-inflammatory and lipid-regulating properties, making it a promising candidate for chronic disease prevention [[2], [3], [4], [5], [6], [7]] and as an adjunct in cancer therapy. Currently, vitamin E is mainly produced through chemical synthesis and plant extraction. However, chemical synthesis is typically geared towards animal feed additives and faces challenges in producing specific vitamin E isomers, while plant extraction is constrained by limited resources and high costs [8]. Therefore, developing an efficient and economical production method for δ-tocotrienol is a major research focus. Microbial biosynthesis is emerging as a promising strategy for δ-tocotrienol production due to its economic and environmental benefits, offering a sustainable alternative to traditional methods [9,10].

**Fig. 1:**
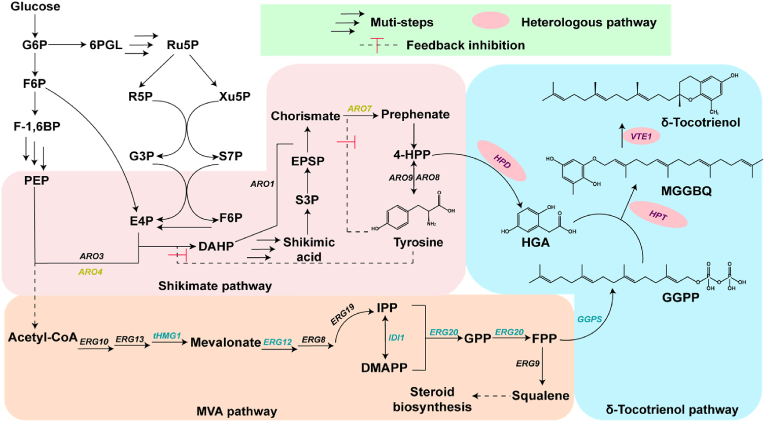
Biosynthetic pathway of δ-tocotrienol in *Y. lipolytica*. The δ-tocotrienol biosynthetic pathway is divided into three modules: the shikimate pathway (pink box), MVA pathway (beige box) and δ-tocotrienol biosynthesis pathway (blue box). Enzymes and intermediates involved include: ARO3, 3-deoxy-7-phosphoheptulonate synthase; ARO4, 3-deoxy-7-phosphoheptulonate syn-thase; ARO1, pentafunctional AROM polypeptide; ARO7, chorismite mutase; ARO8, aromatic aminotransferase I; ARO9, aromatic aminotransferase II; ERG10, acetyl-CoA C-acetyltransferase; ERG13, 3-hydroxy-3-methylglutaryl-CoA (HMG-CoA) synthase; tHMG1, truncated HMG-CoA reductase; ERG12, mevalonate kinase; ERG8, phosphomevalonate kinase; ERG19, diphosphomevalonate decarboxylase; IDI1, isopentenyl diphosphate delta-isomerase; ERG20, farnesyl pyrophosphate synthetase; GGPS, geranylgeranyl diphosphate synthase; HPD, hydroxyphenylpyruvate dioxygenase; HPT, homogentisate phytyl transferase; VTE1, tocopherol cyclase; G6P, glucose-6-phosphate; 6PGL, 6-phosphoglucono-δ-lactone; Ru5P, ribulose 5-phosphate; R5P, ribose-5-phosphate; Xu5P, d-xylulose 5-phosphate; G3P, 3-phosphoglycerate; S7P, sedoheptulose-7-phosphate; F6P, fructose-6-phosphate; F-1,6BP, fructose-1,6-bisphosphate; PEP, phosphoenolpyruvate; E4P, erythrose-4-phosphate; DAHP, 3-deoxy-arabino-heptulonate-7-phosphate; 4-HPP, 4-hydroxyphenylpyruvate; IPP, isopentenyl diphosphate; DMAPP, dimethylallyl diphosphate; GPP, geranyl pyrophosphate; FPP, farnesyl diphosphate; GGPP, geranylgeranyl pyrophosphate; HGA, homogentisic acid; MGGBQ, 2-methyl-6-geranylgeranyl benzoquinol.

The biosynthetic pathway of δ-tocotrienol, which has been well clarified (Fig. 1), involves two critical precursors: homogentisic acid (HGA) from the shikimate pathway and geranylgeranyl pyrophosphate (GGPP) from the mevalonate (MVA) pathway [11]. These precursors undergo a condensation reaction catalysed by homogentisate phytyltransferase (HPT) to form 2-methyl-6-geranylgeranyl-1,4-benzoquinone (MGGBQ), which is subsequently converted to δ-tocotrienol by tocopherol cyclase (VTE1). Previous studies successfully achieved heterologous synthesis of δ-tocotrienol in *Escherichia coli* and *Saccharomyces cerevisiae* [[12], [13], [14]]. For example, Albermann et al. achieved δ-tocotrienol synthesis in *E. coli* by reconstructing and optimising the biosynthetic pathway, yielding 15 μg/g of δ-tocotrienol [12]. In *S. cerevisiae*, the δ-tocotrienol pathway was integrated into the genome and precursor biosynthesis was optimised, yielding 4.1 mg/L of δ-tocotrienol under optimal fermentation conditions [13]. In addition, some metabolic engineering strategies have also been used to further increase the production of δ-tocotrienol. For example, Jiao et al. implemented pathway engineering, the design of transcriptional regulatory factors and *in situ* extraction during fermentation, achieving efficient synthesis and secretion of δ-tocotrienol in *S. cerevisiae* with a titre of 241.7 mg/L in shake flask cultures [14]. However, although some progress has been made, output remains insufficient to meet the needs of industrial production.

Previous studies demonstrated that enhancing precursor availability and boosting the catalytic efficiency of key enzymes are essential for maximising the synthesis of target products [[15], [16], [17], [18]]. For example, Luo et al. achieved high lycopene titres by increasing isopentenyl diphosphate/dimethylallyl diphosphate synthesis efficiency [19]. Guo et al. have successfully achieved the attenuation of the activity of the endogenous lanosterol synthase Erg7P in *S. cerevisiae* through the engineering modification of its critical amino acid sites, thereby considerably enhancing the synthesis efficiency of triterpenoids within the *S. cerevisiae* [20]. Similarly, to ensure the sufficient supply of precursors, the shikimate pathway and the MVA pathway must be subjected to appropriate engineering design, and it is essential to rationally design key enzymes for improved catalytic activity. 10.13039/100014337Furthermore, the oleaginous yeast *Yarrowia lipolytica* has a cytoplasmic abundance of acetyl-CoA and nicotinamide adenine dinucleotide phosphate, which supports downstream synthesis [[21], [22], [23], [24]]. Additionally, its intracellular liposomes offer a hydrophobic environment favourable for the synthesis and storage of hydrophobic natural products such as fat-soluble δ-tocotrienol [25,26]. Therefore, the present study aimed to construct the biosynthetic pathway of δ-tocotrienol in *Y. lipolytica* and optimise the pathway through metabolic engineering and enzyme engineering to achieve efficient synthesis of δ-tocotrienol.

To construct and optimise the δ-tocotrienol biosynthesis pathway in *Y. lipolytica*, we first screened and expressed δ-tocotrienol biosynthesis genes from different sources, creating a strain with an δ-tocotrienol titre of 35.1 mg/L. Subsequently, several strategies were implemented to enhance the catalytic efficiency of rate-limiting steps in the pathway, including the fusion of key enzymes SyHPT and AtVTE1 via a rigid/flexible linker to construct substrate-catalytic channels, semi-rational design of SyHPT to optimise its catalytic activity and multi-copy integration of pathway genes. By combining these strategies with fermentation optimisation, we ultimately achieved a final δ-tocotrienol titre of 466.8 mg/L in a 5 L bioreactor. This study provides insights for synthesising vitamin E and other natural products in *Y. lipolytica*, highlighting its potential as a novel platform for high-value compound production.

## 2 Materials and methods

### 2.1 Strains and Media

The starting strain, *Y. lipolytica* Δku70, was derived by knocking out the *Ku70* gene from the Po1f strain [27]. All primers used in this study are listed in Table S3. *E. coli* JM109 was used for plasmid construction and amplification, cultured in Luria-Bertani (LB) broth or on LB agar plates with 100 mg/L ampicillin at 37 °C. *Y. lipolytica* was cultured on yeast nitrogen base (YNB) medium (6.74 g/L YNB, with or without 5 g/L amino acids, and 20 g/L glucose) or yeast extract peptone dextrose (YPD) medium (20 g/L glucose, 20 g/L peptone, and 10 g/L yeast extract) at 30 °C.

### 2.2 Construction of plasmids and strains

The strains and plasmids used in this study for the biosynthesis of δ-tocotrienol are shown in Tables S1 and S2. Genes related to the biosynthesis of δ-tocotrienol were integrated into the genome of the *Y. lipolytica* Δku70 strain through homologous recombination. These exogenous or endogenous genes were controlled by the TEF promoter (P_TEF_) and XPR2 terminator (T_XPR2_) and were assembled using a Gibson Assembly Kit (Wuxiweiwo, Wuxi, China). All exogenous genes were synthesised by Azenta Biotechnology (Suzhou, China), while endogenous genes were amplified from the *Y. lipolytica* Δku70 genome using Phusion High-Fidelity DNA Polymerase (Vazyme Biotechnology, Nanjing, China). CRISPR-Cas9 plasmids (containing the specific guide RNA and leucine marker) and linearized homologous donor plasmids were used for single-copy site-directed integration of a specific gene, and the specific guide RNA was designed using the CRISPRRRGEN tool (http://www.rgenome.net/cas-designer/). The transformation of *Y. lipolitica* was conducted according to the instructions provided in the Frezen-EZ Yeast Transformation II Kit (Zymo Research, CA, USA). After transformation, cultures were spread on selective solid medium and incubated for 3–5 days at 30 °C. For multi-copy integration, 26S ribosomal DNA (rDNA) and zeta sequences were amplified from the *Y. lipolytica* genome. We referred to the method previously established by Liu et al. [28] for the integration strategy of multi-copy sites. These gene fragments were linked to the upstream and downstream homologous arms of the 26S rDNA or zeta sequences, carrying the *URA3* or *LEU2* selection markers, forming integrative plasmids for these multi-copy sites. The desired target fragments were linearized for yeast transformation using the method of Liu et al., and cells were spread on selective medium (with *URA3* or *LEU2* marker) and incubated for 3−5 days at 30 °C.

### 2.3 Strain Cultivation and fed-batch fermentation

All small-scale fermentations were conducted in 250 mL shake flasks. First, single colony was picked from YPD plates incubated for 2−3 days at 30 °C, inoculated into 5 mL YPD medium in a 50 mL centrifuge tube, and incubated at 30 °C with shaking at 220 rpm for 24 h. Cultures were then transferred to 250 mL shake flasks containing 25 mL YPD medium for fermentation. This study also compared the ability of extractants from different sources to capture δ-tocotrienol, and the results showed that olive oil was effective. Since olive oil does not contain δ-tocotrienol, 10 % olive oil was added to the medium after 24 h of fermentation to capture δ-tocotrienol (Figure S1, S2 and S3).

For fed-batch fermentation, strain was first cultured on YPD plates for 2−3 days at 30 °C. A single colony was inoculated into 10 mL YPD medium in a 50 mL centrifuge tube, incubated at 30 °C and 220 rpm for 24 h, then transferred (5 % inoculum) into a 200 mL YPD medium shake flask and cultured for an additional 24 h. This seed culture was inoculated into a 5 L bioreactor at an initial OD_600_ of 0.6–0.8. Fermentation was conducted at 28 °C for 120 h with 2.5 L of modified YPD medium (40 g/L tryptone, 20 g/L yeast extract, 40 g/L glucose, essential metals, vitamins, and 75 mg/L FeSO_4_). The trace metal solution and the vitamin solution were modified according to the previous report by Sun et al. [29]. The trace metal solution consisted of 4.5 g/L ZnSO_4_·7H_2_O, 4.5 g/L CaCl_2_·2H_2_O, 3 g/L FeSO_4_·7H_2_O, 1 g/L CuCl_2_·2H_2_O, 1 g/L H_3_BO_3_, 0.4 g/L Na_2_MoO_4_·2H_2_O, 0.3 g/L CoCl_2_·6H_2_O, 0.1 g/L CuSO_4_·5H_2_O, 0.1 g/L KI and 15 g/L EDTA. The pH was adjusted to 4.0. The vitamin solution consisted of 0.05 g/L biotin, 0.2 g/L *p*-aminobenzoic acid, 1 g/L nicotinic acid, 1 g/L calcium pantothenate, 1 g/L pyridoxine phosphate, 1 g/L thiamine HCl and 25 g/L *myo*-inositol. Fed-batch fermentation was conducted under the following conditions: the temperature of fermentation was 28 °C, ammonium hydroxide was supplied to maintain the pH at 5.0 during the fermentation, an agitation cascade ranging from 300 to 900 rpm was used to regulate the dissolved oxygen at 20 %. Glucose levels were maintained below 1 g/L throughout fermentation.

### 2.4 Product extraction and Analytical methods

To measure HGA, 1 mL of fermentation broth was centrifuged at 12,000 *g* for 10 min, and the supernatant was filtered through a 0.22 μm filter for high-performance liquid chromatography (HPLC) analysis. For δ-tocotrienol extraction, 10 % olive oil was added to the medium, and the sample was centrifuged at 12,000 *g* for 5 min to separate organic and aqueous layers. The organic layer was centrifuged again, and 100 μL was diluted in 900 μL dimethyl sulphoxide, filtered, and analysed by HPLC. HGA detection was conducted on an LC-20AT HPLC system (Shimadzu, Kanda Nishiki-cho, Chiyoda-ku, Tokyo) with a variable wavelength detector and a ZORBAX Eclipse XDB-C18 column (Agilent, California, America). The mobile phase was a water-acetonitrile mixture containing 0.1 % trifluoroacetic acid, at a 1 mL/min flow rate and a 10 μL injection volume. A binary high-pressure gradient elution was maintained over 25 min, with detection at 292 nm. For δ-tocotrienol detection, the same HPLC system and column were used. The mobile phase consisted of water and acetonitrile with 0.1 % trifluoroacetic acid, with pump A set at 0.02 mL/min and pump B at 0.98 mL/min. A 10 μL injection was analysed via isocratic elution over 26 min, with detection at 280 nm.

The identification of δ-tocotrienol was conducted using a Waters MALDI SYNAPT Q-TOF mass spectrometry system (Waters, MA, USA) in a positive ion mode (the LC-Q-TOF/MS spectra of δ-tocotrienol standard and the sample are shown in Fig. S2). The column temperature was maintained at 50 °C, and the volume was 10 μL. The liquid chromatography conditions were consistent with those of the HPLC method.

## 3 Results

### 3.1 Construction of the δ-tocotrienol biosynthetic pathway in *Y. lipolytica*

**Fig. 2:**
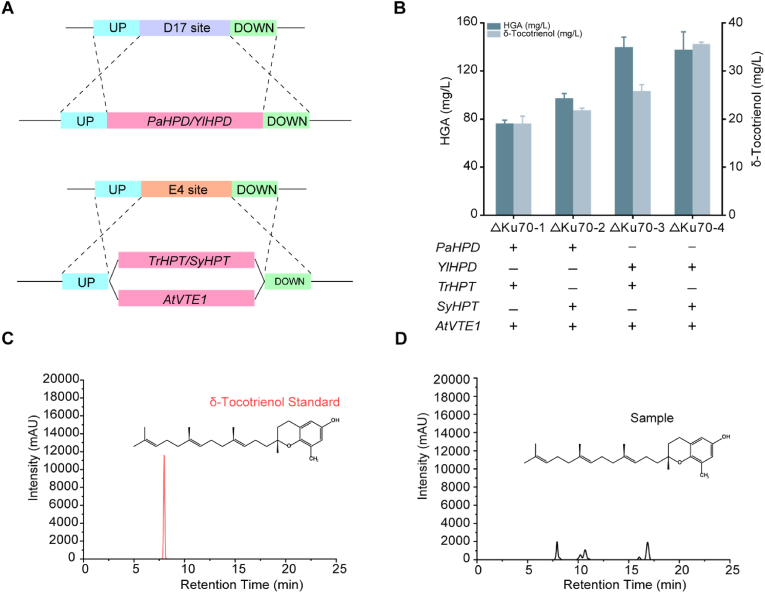
Expression of δ-tocotrienol biosynthesis pathway enzymes from various sources. (A) Gene integration at distinct loci: HGA synthesis genes at the D17 locus; MGGBQ and δ-tocotrienol synthesis genes at the E4 locus. (B) Effects of expressing δ-tocotrienol biosynthesis pathway genes from different sources. "+" denotes gene integration into the chromosome; "—" denotes no integration. (C) HPLC chromatogram of the δ-tocotrienol standard. (D) HPLC chromatogram of the fermentation broth. Values are averages of three independent experiments. Error bars indicate standard deviation.

The biosynthesis of δ-tocotrienol requires two precursors: HGA from the shikimate pathway and GGPP from the MVA pathway. For HGA synthesis, 4-hydroxyphenylpyruvate (4-HPP) is generated by the endogenous enzyme 4-hydroxyphenylpyruvate dioxygenase (HPD) in *Y. lipolytica*. Studies have also indicated that HGA can be efficiently produced in *S. cerevisiae* using PaHPD from *Pseudomonas putida*. Regarding the selection of HPT, we chose SyHPT from *Synechocystis* sp. and *Triticum aestivum* to catalyse the formation of MGGBQ from HGA and GGPP. Meanwhile, AtVTE1 from *Arabidopsis thaliana* was chosen to catalyse the formation of δ-tocotrienol from MGGBQ. To construct the δ-tocotrienol biosynthesis pathway, different combinations of these genes were introduced into *Y. lipolytica*, enabling us to screen for the optimal gene combination. This process ultimately resulted in the construction of strains Δku70-1 to Δku70-4. *PaHPD/YlHPD* were integrated into the D17 locus of the *Y. lipolytica* genome, while *TrHPT/SyHPT* and *AtVTE1* were integrated into the E4 locus (Fig. 2A). Liquid chromatography-mass spectrometry (LC-MS) analysis confirmed δ-tocotrienol production across all strains (Fig. 2C and. D and Figure S4). Among the gene combinations tested, *YlHPD-SyHPT-AtVTE1* exhibited the highest δ-tocotrienol titre of 35.1 mg/L (Fig. 2B), leading to the selection of the Δku70-4 strain for further metabolic engineering.

### 3.2 Engineering the shikimate pathway to enhance the supply of HGA

**Fig. 3:**
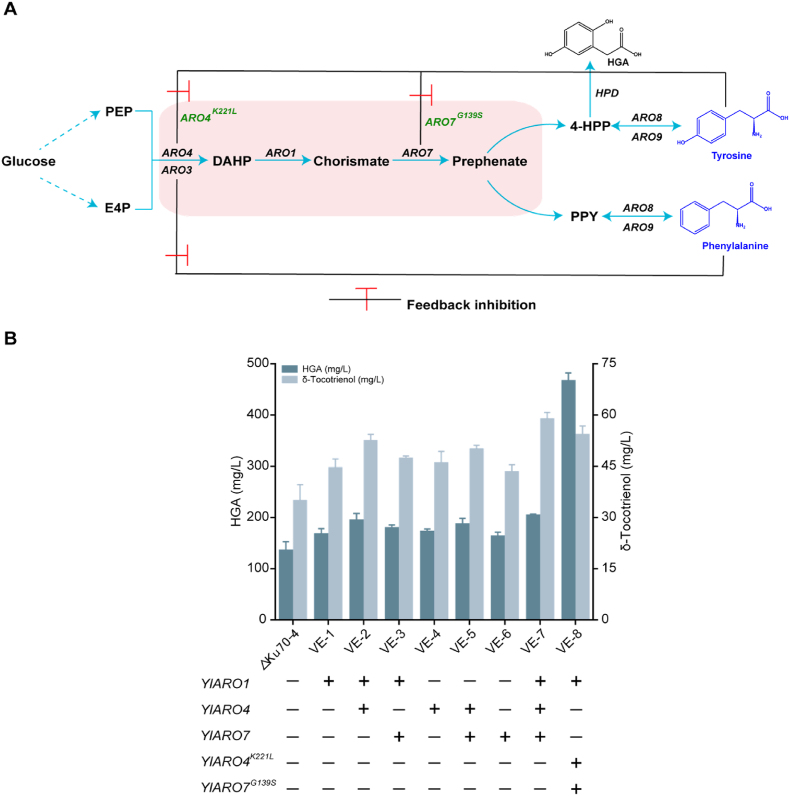
Effect of enhancing the shikimic acid pathway on the synthesis of δ-tocotrienol. (A) Schematic diagram of the HGA biosynthesis pathway in *Y. lipolytica*. PEP, phosphoenolpyruvate; E4P, erythrose-4-phosphate; DAHP, 3-deoxy-arabino-heptulonate-7-phosphate; 4-HPP, 4-hydroxyphenylpyruvate; PPY, phenylpyruvate; HGA, homogentisic acid. (B) Combinatorial expression of the genes *ARO1*, *ARO4*, *ARO7*, *ARO4*^*K221L*^ and *ARO7*^*G139S*^ in *Y. lipolytica*. Values are averages of three independent experiments. Error bars indicate standard deviation.

HGA, a precursor in δ-tocotrienol biosynthesis, originates from the endogenous shikimate pathway in *Y. lipolytica*. 3-deoxy-7-phosphoheptulonate synthase (ARO4) catalyses the condensation of phosphoenolpyruvate (PEP) and erythrose 4-phosphate (E4P) to form 3-deoxy-2-arabino-heptulosonate-7-phosphate (DAHP), which is converted into branched-chain acids by Aro1 and further into prephenate by Aro7. Finally, prephenate is converted into 4-HPP and subsequently into HGA by HPD (Fig. 3A). However, these processes are subject to tyrosine-induced feedback inhibition. Previous studies reported that overexpression of the mutant genes *ARO4*^*K221L*^ and *ARO7*^*G139S*^ can relieve this feedback inhibition [23], increasing the flux of the shikimate pathway.

We overexpressed *ARO1*, *ARO4*, *ARO7*, *ARO4*^*K221L*^ and *ARO7*^*G139S*^ in different combinations in the Δku70-4 strain, resulting in strains VE-1 to VE-8 (Fig. 3A). Each combination achieved higher HGA and δ-tocotrienol titres than Δku70-4, with VE-7 exhibiting the highest δ-tocotrienol titre at 59.2 mg/L (Fig. 3B). Notably, VE-8, which overexpressed *ARO4*^*K221L*^ and *ARO7*^*G139S*^, achieved an HGA titre of 463.8 mg/L but a lower δ-tocotrienol titre of 54.4 mg/L, suggesting that excessive HGA may not be required for δ-tocotrienol synthesis, and that GGPP availability might limit production. Therefore, VE-7 was selected for further optimisation.

### 3.3 Enhancing MVA pathway flux to increase the supply of GGPP

**Fig. 4:**
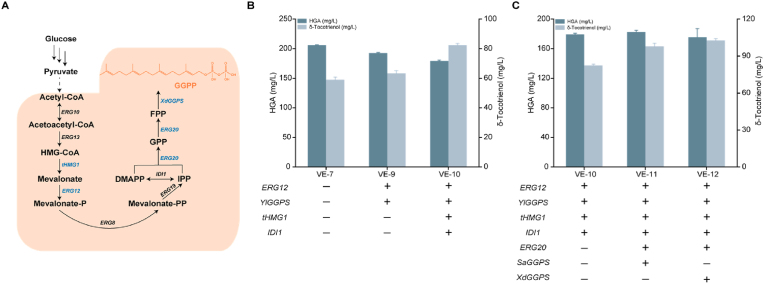
Effect of enhancing the MVA pathway on the synthesis of δ-tocotrienol. (A) Schematic diagram of GGPP synthesis via the endogenous MVA pathway in *Y. lipolytica*. HMG-CoA, 3-hydroxy-3-Menthyl-Glutaryl-CoA; IPP, isopentenyl diphosphate; DMAPP, dimethylallyl diphosphate; GPP, geranyl pyrophosphate; FPP, farnesyl diphosphate; GGPP, geranylgeranyl pyrophosphate. (B) Overexpression of key genes in the MVA pathway (*RG12*, *YlGGPS*, *tHMG1* and *IDI1*). "+" indicates that the gene has been integrated into the strain's chromosome, while "—" indicates that the gene has not been integrated. (C) Comparison of the effects of GGPP synthases from *Sulfolobus acidocaldarius* and *Xanthophyllomyces dendrorhous* on the synthesis of δ-tocotrienol. "+" indicates that the gene has been integrated into the strain's chromosome, while "—" indicates that the gene has not been integrated. Values are averages of three independent experiments. Error bars indicate standard deviation.

GGPP is a critical precursor in δ-tocotrienol biosynthesis, synthesised via the MVA pathway in *Y. lipolytica*. Within this pathway, acetyl-CoA is converted through sequential steps into farnesyl pyrophosphate (FPP), which is then converted into GGPP by GGPP synthase (Fig. 4A). Studies have identified HMG-CoA synthase genes HMGR (HMG1 and HMG2) as key bottlenecks in the MVA pathway. Additionally, overexpressing a truncated HMGR gene (*tHMG1*) reduces feedback inhibition by sterol compounds, enhancing FPP accumulation. Therefore, we overexpressed some key MVA pathway genes including mevalonate kinase (*ERG12*), geranylgeranyl diphosphate synthase (*GGPS*), *tHMG1* and isopentenyl diphosphate delta isomerase (*IDI1*), resulting in an increase in the titre of δ-tocotrienol to 82.4 mg/L (VE-10 strain), a 39 % improvement compared with the VE-7 strain (Fig. 4B).

To further enhance the supply of GGPP, we expressed the endogenous genes farnesyl pyrophosphate synthetase (*ERG20*), *SaGGPS* and *XdGGPS* from *Y. lipolytica* in the VE-10 strain, generating strains VE11 and VE12. Compared with the VE-10 strain, co-expression of GGPP synthase genes from *Sulfolobus acidocaldarius* and *Xanthophyllomyces dendrorhous* resulted in δ-tocotrienol titres of 97.8 mg/L and 102.8 mg/L, representing increases of 19 % and 25 %, respectively (Fig. 4C). Therefore, enhancing the flux of the MVA pathway can improve the supply of GGPP, facilitating the synthesis of δ-tocotrienol.

### 3.4 Expression of a *SyHPT*-*AtVTE1* fusion

**Fig. 5:**
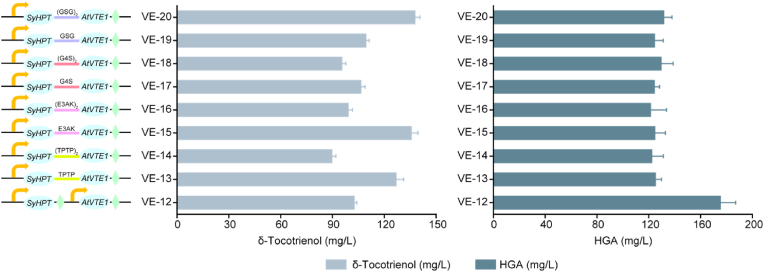
Effect of linker type in *SyHPT-AtVTE1* fusions on δ-tocotrienol biosynthesis. Fusion protein linkers TPTP, (TPTP)_2_ (TPTPTPTP), E3AK (EAAAK), (E3AK)_2_ (EAAAKEAAAK), G4S (GGGGS), (G4S)_2_ (GGGGSGGGGS), GSG and (GSG)_2_ (GSGGSG) were tested. Values are averages of three independent experiments. Error bars indicate standard deviation.

Linking two proteins is an excellent strategy for forming unnatural fusions that combine the functions of biological enzymes and catalytic activities. An appropriate linker can maintain the optimal distance between catalytic centres, thereby enhancing catalytic efficiency. Therefore, we tested *SyHPT*-*AtVTE1* fusion proteins linked by rigid linkers TPTP, (TPTP)_2_, EAAAK and (EAAAK)_2_), and flexible linkers GGGGS, (GGGGS)_2_, GSG and (GSG)_2_), to improve δ-tocotrienol production in VE-12, resulting in the generation of strains VE-13 to VE-20. The results showed that fusion proteins with TPTP, GGGGS, (GSG)_2_, GSG and EAAAK linkers achieved enhanced δ-tocotrienol titres, with VE-20 expressing the fusion protein with a (GSG)_2_ linker achieving the highest titre of 137.9 mg/L (Fig. 5). This increase in catalytic efficiency is likely due to the close interaction between the catalytic centres of SyHPT and AtVTE1, which depends on optimal spacing. By using linkers to fuse SyHPT and AtVTE1, the active sites are brought closer together, thereby enhancing enzyme efficiency. Consequently, we selected the VE-20 strain for the next round of modifications.

### 3.5 Semi-rational design of *SyHPT*

**Fig. 6:**
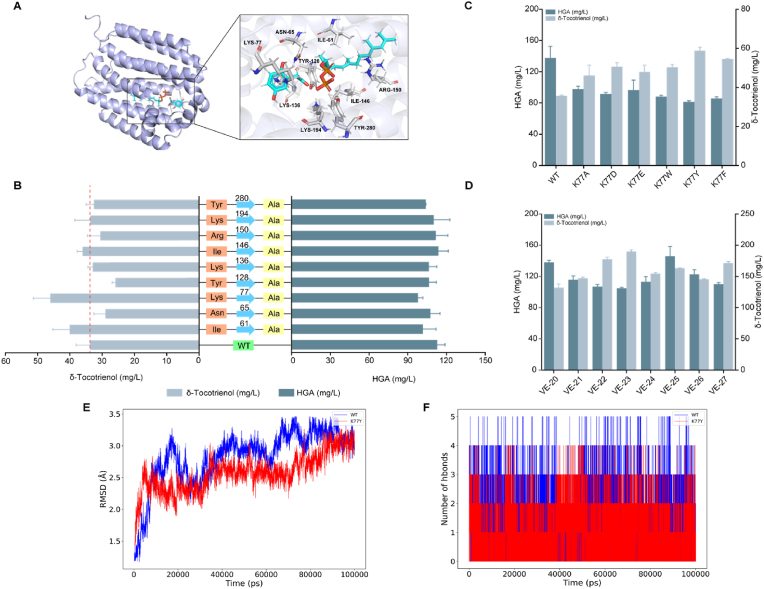
Structure of the SyHPT protein and preliminary mutation results. (A) Predicted structure of the SyHPT protein and amino acid residues in its active site pocket. Substrates are represented by blue sticks and amino acid residues by grey sticks. (B) Relative activity of alanine scanning mutants of SyHPT. Values are averages of three independent experiments. Error bars indicate standard deviation. (C) Mutation of lysine 77 to acidic and aromatic amino acids. Values are average of three independent experiments. Error bars indicate standard deviation. (D) Integration of SyHPT^K77Y^ and AtVTE1 mutants into the multi-copy sites of *Yarrowia lipolytica*, and complementation with *LEU2* and *URA3* markers. Values are averages of three independent experiments. Error bars indicate standard deviation. (E) Root mean squared deviation of atomic positions for WT (blue) and SyHPT^K77Y^ (red) proteins in 100 ns molecular dynamics (MD) simulations. (F) Number of hydrogen atoms in WT (blue) and SyHPT^K77Y^ (red) proteins.

In the δ-tocotrienol biosynthesis pathway, HPT and VTE1 are two rate-limiting enzymes that limit the synthesis and conversion of MGGBQ. To improve the catalytic activity of SyHPT, we firstly used AlphaFold 3 [30] to predict its protein structure (Fig. 6A), and docked HGA and GGPP into its substrate-binding pocket using Discovery Studio [31], resulting in structure models of enzyme-substrate complexes. Based on analysis of these structural models, amino acid residues including I61, N65 and K77 within 5 Å of the substrate-binding pocket that may interact with the substrate were selected as target sites for subsequent modifications. These residues were subjected to alanine scanning mutagenesis. The catalytic activity of the I61A, K77A and I146A mutants was significantly increased, by 19 %, 36 % and 7 %, respectively, compared with wild-type (WT) SyHPT (Fig. 6B). Therefore, lysine at position 77 was selected for further mutation to acidic amino acids (aspartic acid, glutamic acid), and amino acids with side chains containing a benzene ring (tryptophan, tyrosine and phenylalanine), to enhance the enzyme's proton-capturing ability and its interaction with the substrate molecule. Experimental results showed that mutating lysine to tyrosine further increased the titre" of δ-tocotrienol by 27 % compared to SyHPT^K77A^ and by 67 % compared to the WT strain (Fig. 6C).

**Fig. 7:**
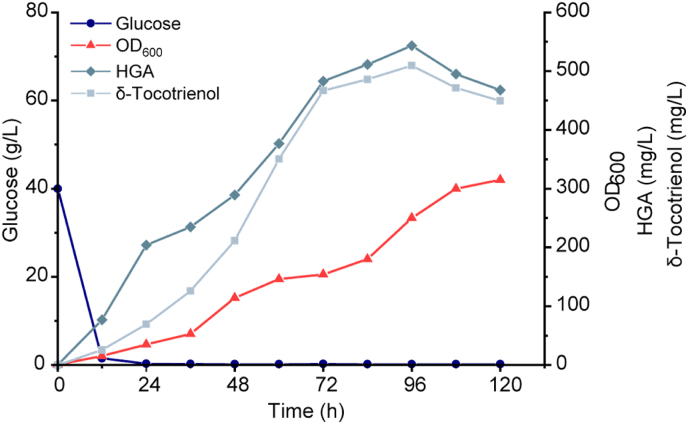
Fermentation of δ-tocotrienol by strain VE-23 in a 5 L bioreactor. Time courses are shown for glucose concentration, cell density, δ-tocotrienol and HGA production in fed-batch fermentation in a 5 L bioreactor at pH 5.0 with 20 % dissolved oxygen.

To elucidate the catalytic mechanism of the mutants, we conducted molecular dynamics simulations, which revealed higher structural stability for the SyHPT^K77Y^ mutant, due to a greater number of hydrogen bonds and improved binding free energy (−73.4 kcal/mol compared to −71.7 kcal/mol for WT), indicating higher binding affinity and enzymatic activity (Fig. 6E and F). Subsequently, integration of the K77Y mutant and biosynthetic genes into the multi-copy sites of *Y. lipolytica* yielded a final δ-tocotrienol titre of 189.9 mg/L for strain VE-23 strain (Fig. 6D).

### 3.6 Fed-batch fermentation to enhance δ-tocotrienol production

To further increase the titre of δ-tocotrienol, batch feeding fermentation was performed using the optimal production strain VE-23 in a 5 L bioreactor. During the initial stage of fermentation, the dissolved oxygen level was maintained at around 20 % by controlling the stirring speed and air flow rate. As the fermentation time progressed, the pH of the fermentation broth gradually decreased. Therefore, the pH of the fermentation broth was maintained at around 5.0 by automatically adding 50 % NH_3_·H_2_O. When glucose was depleted, it was fed in batches. The feeding rate of glucose was 12–18 mL/h, so that the glucose concentration in the fermentation broth was always kept at 0.3–0.8 g/L until the end of fermentation. The fermentation results showed that HGA and δ-tocotrienol had a significant accumulation in the early stage of fermentation. The accumulation of δ-tocotrienol was around 200 mg/L after 48 h. As the fermentation time went on, the titre of δ-tocotrienol gradually increased. After 96 h, 466.8 mg/L of δ-tocotrienol was accumulated (Fig. 7), the highest titre for δ-tocotrienol production reported in microorganisms to date (Table 1).

**Table 1:** Tocotrienols biosynthesis by microbial cell factories.

chassis	product	titre	fermentation mode	strategy	references
*E. coli*	δ-tocotrienol	15 μg/g DCW	in shake flasks	overexpression of heterologous	[12]
*S. cerevisiae*	δ-tocotrienol	4.10 ± 0.10 mg/L	In 2L fermenter	overexpression of heterologous; optimisation of the fermentation	[13]
	α, β, γ, δ-tocotrienol	82.68 mg/L	in shake flasks	addition of olive oil to promote the efflux of tocotrienols	[32]
	α, β, γ, δ-tocotrienol	320 mg/L	in 5 L fermenter	truncation of the transit peptide; development of a cold-shock-triggered temperature control system	[33]
	δ-tocotrienol	241.7 mg/L	in shake flasks	strengthening the shikimate pathway and the MVA pathway; addition of 2-hydroxypropyl-β-cyclodextrin	[14]
*Y. lipolitica*	δ-tocotrienol	466.8 mg/L	in 5 L fermenter	fusion of SyHPT and AtVTE1; semi-rational design of SyHPT	This study

## 4 Discussion

Compared with traditional plant extraction and chemical synthesis methods, constructing microbial cell factories for the biosynthesis of δ-tocotrienol is an efficient, environmentally friendly, and economical alternative [[34], [35], [36]]. However, biosynthesis of δ-tocotrienol is limited by insufficient precursor supply and low catalytic efficiency of key enzymes. This study achieved the *de novo* synthesis of δ-tocotrienol in *Y. lipolytica* by reengineering its metabolic pathway for the first time. Additionally, strategies were adopted to improve the δ-tocotrienol synthesis efficiency, including strengthening the MVA pathway and the shikimic acid pathway to supply two essential precursors (HGA and GGPP), fusion expression of key enzymes with rigid and flexible linkers to construct substrate catalytic channels, enhancing the catalytic efficiency of the key enzyme SyHPT, and fermentation process optimisation, ultimately resulting in a δ-tocotrienol titre of 469.8 mg/L. Overall, these strategies may have broad applications in the microbial synthesis of vitamin E-related compounds.

Efficient precursor synthesis is essential for maximising target compound yields [[37], [38], [39]]. Several studies have reported the efficient synthesis of target products by improving the synthesis efficiency of precursors. For example, Wegner et al. enhanced the supply of acetyl-CoA to increase the availability of MVA in *S. cerevisiae* by inhibiting the expression of squalene synthase (ERG9), overexpressing pantothenate kinase CAB1, and supplementing pantothenic acid, resulting in a 360-fold increase in MVA content in yeast [40]. Biosynthesis of δ-tocotrienol requires two essential precursors, HGA and GGPP, whose intracellular synthesis efficiency directly affects the δ-tocotrienol accumulation level. In this study, we improved the synthesis efficiency of HGA by overexpressing key genes in the shikimate pathway and introducing tyrosine feedback inhibition-resistant mutants [[41], [42], [43], [44]], *ARO4*^*K221L*^ and *ARO7*^*G139S*^, which significantly increased the titre of δ-tocotrienol. However, we observed that limited availability of GGPP was a bottleneck that constrained further improvements in δ-tocotrienol synthesis efficiency. To address this, we enhanced GGPP synthesis by overexpressing key genes in the MVA pathway and screening various GGPP synthase sources, which further promoted δ-tocotrienol accumulation.

When constructing a synthetic pathway for a target product involving multiple genes, several challenges may arise. For instance, pathway intermediates can be diverted by endogenous reactions within the host strain or lost through secretion. Additionally, rapid diffusion and degradation of intermediates may lead to toxicity for the host. Fusion expression of key pathway enzymes is an effective strategy to address these issues [[45], [46], [47]]. This approach enhances the local concentration of pathway enzymes and metabolites by spatially organising enzymes into multi-enzyme complexes, thereby facilitating substrate channelling, improving enzyme conversion efficiency and reducing intermediate accumulation. For example, Liu et al. demonstrated that fusion expression of the key enzymes 4-coumarate-CoA ligase and resveratrol synthase for resveratrol production using rigid and flexible linkers increased the resveratrol titre by 1.4-fold [23]. In the present study, different linkers were screened to optimise the spatial organisation of the key enzymes SyHPT and AtVTE1 in the δ-tocotrienol biosynthesis pathway to form a substrate channel, which ultimately increased the δ-tocotrienol titres by 34 %. Therefore, the strategy of fusing key enzymes into multi-enzyme complexes holds significant potential for enhancing the synthesis of target products.

The activity, stability and specificity of natural enzymes directly influence the overall efficiency of cell factories, making enzyme performance a primary bottleneck in enhancing cell factory productivity [48]. To address limitations in natural enzyme activity, various strategies have been employed to improve enzyme performance including rational design [49], directed evolution [50], semi-rational design [51] and artificial intelligence-assisted design [52]. For example, Gong et al. (2024) achieved the production of mogroside III through structure-guided directed evolution and combinatorial active site saturation mutagenesis [53]. In the present study, in addition to enhancing the precursor supply of δ-tocotrienol, we employed a semi-rational design strategy to enhance the catalytic activity of the key enzyme SyHPT, the critical site was identified by alanine scanning mutagenesis. Then, by mutating this site into other acidic or benzene-ring-containing side-chain amino acids, the K77Y mutant was found, achieving a 67 % increase in δ-tocotrienol titre compared to WT. Molecular dynamics simulations further revealed that the enhanced activity of the mutant is due to its more stable protein structure and reduced binding free energy. Thus, enhancing the catalytic activity of key enzymes is an important strategy for improving cell factory efficiency when synthesising target products.

In summary, this study successfully established a biosynthetic pathway for δ-tocotrienol in *Y. lipolytica* for the first time. Efficient biosynthesis of δ-tocotrienol was achieved by enhancing the shikimate and MVA pathways to improve precursor supply, employing a substrate channel engineering strategy by co-expressing key enzymes, applying semi-rational strategies to enhance key enzyme activities and optimising fermentation processes. Under optimal conditions, accumulation of δ-tocotrienol reached 466.8 mg/L, the highest titre of δ-tocotrienol production reported in microorganisms to date. The findings offer a valuable reference for the synthesis of vitamin E-related compounds and their potential for industrial production.

## CRediT authorship contribution statement

**Jinbo Xiang:** Writing – original draft. **Mengsu Liu:** Methodology. **Xinglong Wang:** Methodology. **Mingyu Yue:** Methodology. **Zhijie Qin:** Funding acquisition. **Jingwen Zhou:** Writing – review & editing, Resources, Methodology.

## Declaration of competing interest

The authors declare that they have no known competing financial interests or personal relationships that could have appeared to influence the work reported in this paper.
