# Supplementary material for: Combined metabolic and enzymatic engineering for de novo biosynthesis of δ-tocotrienol in Yarrowia lipolytica
Source: Synth Syst Biotechnol. 2025 Feb 20;10(3):719–27. doi: 10.1016/j.synbio.2025.02.011 (PMC12002712; doi:10.1016/j.synbio.2025.02.011)
Supplement: Multimedia component 1 [file mmc1.docx]

**Supplementary Materials**

**Combined metabolic and enzymatic engineering for *de novo* biosynthesis of** **δ-tocotrienol in *Yarrowia lipolytica***

Jinbo Xiang^1,2^, Mengsu Liu^1,2^, Xinglong Wang^1,2,^, Mingyu Yue^1,2,^, Zhijie Qin^1,2^, Jingwen Zhou^1,2,3*^

^1^ Engineering Research Center of Ministry of Education on Food Synthetic Biotechnology, School of Biotechnology, Jiangnan University, 1800 Lihu Road, Wuxi, Jiangsu 214122, China;

^2^ Science Center for Future Foods, Jiangnan University, 1800 Lihu Rd, Wuxi, Jiangsu 214122, China;

^3^ Jiangsu Province Engineering Research Center of Food Synthetic Biotechnology, Jiangnan University, 1800 Lihu Road, Wuxi, Jiangsu 214122, China.

^*^ Correspondence authors

Jingwen Zhou

Mailing address: Science Center for Future Foods, Jiangnan University, 1800 Lihu Road, Wuxi, Jiangsu 214122, China

Phone: +86-510-85914371; Fax: +86-510-85914371

^*^ E-mail: [jlu@jiangnan.edu.cn](mailto:jlu@jiangnan.edu.cn); zhoujw1982@jiangnan.edu.cn

**Supplementary Table**

**Table S****1. strains used in this study**

| **Strains** | **Description** | **Source** |
| --- | --- | --- |
| JM109  Polf  Δku70  Δku70-1  Δku70-2  Δku70-3  Δku70-4  VE-1  VE-2  VE-3  VE-4  VE-5  VE-6  VE-7  VE-8  VE-9  VE-10  VE-11  VE-12  VE-13  VE-14  VE-15  VE-16  VE-17  VE-18  VE-19  VE-20  K77A  K77A/I61A  K77A/I146A  K77A/I61A/I146A  K77D  K77E  K77W  K77Y  K77F  VE-21  VE-22  VE-23  VE-24  VE-25  VE-26 | Cloning strain  *Y. lipolitica* W29, MATa, URA3-302, LEU2-270, XPR2-322, AXP-2  Polf, *Δku70*  Δku70, D17:: *P_TEF_*-*PaHPD*-*T_XPR2_*; E4:: *P_TEF_*-*TrHPT*-*T_XPR2_*-*P_TEF_*-*AtVTE1*-*T_XPR2_*  Δku70, D17:: *P_TEF_*-*PaHPD*-*T_XPR2_*; E4:: *P_TEF_*-*SyHPT*-*T_XPR2_*-*P_TEF_*-*AtVTE1*-*T_XPR2_*  Δku70, D17:: *P_TEF_*-*YlHPD*-*T_XPR2_*; E4:: *P_TEF_*-*TrHPT*-*T_XPR2_*-*P_TEF_*-*AtVTE1*-*T_XPR2_*  Δku70, D17:: *P_TEF_*-*YlHPD*-*T_XPR2_*; E4:: *P_TEF_*-*SyHPT*-*T_XPR2_*-*P_TEF_*-*AtVTE1*-*T_XPR2_*  Δku70-4, A3:: *P_TEF_*-*ARO1*-*T_XPR2_*  Δku70-4, A3:: *P_TEF_*-*ARO1*-*T_XPR2_*-*P_TEF_*-*ARO4*-*T_XPR2_*  Δku70-4, A3:: *P_TEF_*-*ARO1*-*T_XPR2_*-*P_TEF_*-*ARO7*-*T_XPR2_*  Δku70-4, A3:: *P_TEF_*-*ARO4*-*T_XPR2_*  Δku70-4, A3:: *P_TEF_*-*ARO4*-*T_XPR2_*-*P_TEF_*-*ARO7*-*T_XPR2_*  Δku70-4, A3:: *P_TEF_*-*ARO7*-*T_XPR2_*  VE-5, F4:: *P_TEF_*-*ARO1*-*T_XPR2_*  Δku70-4, A3:: *P_TEF_*-*ARO4^K221L^*-*T_XPR2_*-*P_TEF_*-*ARO7^G139S^*-*T_XPR2_*; F4:: *P_TEF_*-*ARO1*-*T_XPR2_*  VE-7, C7:: *P_TEF_*-*ERG12*-*T_XPR2_*-*P_TEF_*-*YLGGPS*-*T_XPR2_*  VE-9, E5:: *P_TEF_*-*tHMG1*-*T_XPR2_*-*P_TEF_*-*IDI 1*-*T_XPR2_*  VE-10, C1:: *P_TEF_*-*ERG20*-*T_XPR2_*-*P_TEF_*-*SaGGPS*-*T_XPR2_*  VE-10, C1:: *P_TEF_*-*ERG20*-*T_XPR2_*-*P_TEF_*-*XdGGPS*-*T_XPR2_*  VE-12, E2:: *P_TEF_*-SyHPT-(TPTP)-*AtVTE1*-*T_XPR2_*  VE-12, E2:: *P_TEF_*-SyHPT-(TPTP)_2_-*AtVTE1*-*T_XPR2_*  VE-12, E2:: *P_TEF_*-SyHPT-(EAAAK)-*AtVTE1*-*T_XPR2_*  VE-12, E2:: *P_TEF_*-SyHPT-(EAAAK)_2_-*AtVTE1*-*T_XPR2_*  VE-12, E2:: *P_TEF_*-SyHPT-(GGGGS)-*AtVTE1*-*T_XPR2_*  VE-12, E2:: *P_TEF_*-SyHPT-(GGGGS)_2_-*AtVTE1*-*T_XPR2_*  VE-12, E2:: *P_TEF_*-SyHPT-(GSG)-*AtVTE1*-*T_XPR2_*  VE-12, E2:: *P_TEF_*-SyHPT-(GSG)_2_-*AtVTE1*-*T_XPR2_*  Δku70, D17:: *P_TEF_*-*YlHPD*-*T_XPR2_*; E4:: *P_TEF_*-*SyHPT^K77A^*-*T_XPR2_*-*P_TEF_*-*AtVTE1*-*T_XPR2_*  Δku70, D17:: *P_TEF_*-*YlHPD*-*T_XPR2_*; E4:: *P_TEF_*-*SyHPT^K77A/I61A^*-*T_XPR2_*-*P_TEF_*-*AtVTE1*-*T_XPR2_*  Δku70, D17:: *P_TEF_*-*YlHPD*-*T_XPR2_*; E4:: *P_TEF_*-*SyHPT^K77A/I146A^*-*T_XPR2_*-*P_TEF_*-*AtVTE1*-*T_XPR2_*  Δku70, D17:: *P_TEF_*-*YlHPD*-*T_XPR2_*; E4:: *P_TEF_*-*SyHPT^K77A/I61A/I146A^*-*T_XPR2_*-*P_TEF_*-*AtVTE1*-*T_XPR2_*  Δku70, D17:: *P_TEF_*-*YlHPD*-*T_XPR2_*; E4:: *P_TEF_*-*SyHPT^K77D^*-*T_XPR2_*-*P_TEF_*-*AtVTE1*-*T_XPR2_*  Δku70, D17:: *P_TEF_*-*YlHPD*-*T_XPR2_*; E4:: *P_TEF_*-*SyHPT^K77E^*-*T_XPR2_*-*P_TEF_*-*AtVTE1*-*T_XPR2_*  Δku70, D17:: *P_TEF_*-*YlHPD*-*T_XPR2_*; E4:: *P_TEF_*-*SyHPT^K77W^*-*T_XPR2_*-*P_TEF_*-*AtVTE1*-*T_XPR2_*  Δku70, D17:: *P_TEF_*-*YlHPD*-*T_XPR2_*; E4:: *P_TEF_*-*SyHPT^K77Y^*-*T_XPR2_*-*P_TEF_*-*AtVTE1*-*T_XPR2_*  Δku70, D17:: *P_TEF_*-*YlHPD*-*T_XPR2_*; E4:: *P_TEF_*-*SyHPT^K77F^*-*T_XPR2_*-*P_TEF_*-*AtVTE1*-*T_XPR2_*  VE-20, zeta:: *P_TEF_*-*SyHPT^K77Y^*-*T_XPR2_*-*P_TEF_*-*AtVTE1*-*T_XPR2_*-*P_TEF_*-*LEU2*-*T_XPR2_*; 26S rDNA:: *P_TEF_*-*URA3*-*T_XPR2_*  VE-20, zeta:: *P_TEF_*-*SyHPT^K77Y^*-*T_XPR2_*-*P_TEF_*-*AtVTE1*-*T_XPR2_*-*P_TEF_*-*LEU2*-*T_XPR2_*; 26S rDNA:: *P_TEF_*-*URA3*-*T_XPR2_*  VE-20, zeta:: *P_TEF_*-*SyHPT^K77Y^*-*T_XPR2_*-*P_TEF_*-*AtVTE1*-*T_XPR2_*-*P_TEF_*-*LEU2*-*T_XPR2_*; 26S rDNA:: *P_TEF_*-*URA3*-*T_XPR2_*  VE-20, zeta:: *P_TEF_*-*SyHPT^K77Y^*-*T_XPR2_*-*P_TEF_*-*AtVTE1*-*T_XPR2_*-*P_TEF_*-*LEU2*-*T_XPR2_*; 26S rDNA:: *P_TEF_*-*URA3*-*T_XPR2_*  VE-20, zeta:: *P_TEF_*-*SyHPT^K77Y^*-*T_XPR2_*-*P_TEF_*-*AtVTE1*-*T_XPR2_*-*P_TEF_*-*LEU2*-*T_XPR2_*; 26S rDNA:: *P_TEF_*-*URA3*-*T_XPR2_*  VE-20, zeta:: *P_TEF_*-*SyHPT^K77Y^*-*T_XPR2_*-*P_TEF_*-*AtVTE1*-*T_XPR2_*-*P_TEF_*-*LEU2*-*T_XPR2_*; 26S rDNA:: *P_TEF_*-*URA3*-*T_XPR2_* | Our lab  Our lab  Our lab  This study  This study  This study  This study  This study  This study  This study  This study  This study  This study  This study  This study  This study  This study  This study  This study  This study  This study  This study  This study  This study  This study  This study  This study  This study  This study  This study  This study  This study  This study  This study  This study  This study  This study  This study  This study  This study  This study  This study |

**Table S2. Plasmids used in this study**

| **Plasmid** | **Description** | **Source** |
| --- | --- | --- |
| pYLXP'  pYLXP'2  pYlVE01  pYlVE02  pYlVE03  pYlVE04  pYlVE05  pYlVE06  pYlVE07  pYlVE08  pYlVE09  pYlVE10  pYlVE11  pYlVE12  pYlVE13  pYlVE14  pYlVE15  pYlVE16  pYlVE17  pYlVE18  pYlVE19  pYlVE20  pYlVE21  pYlVE22  pYlVE23  pYlVE24  pYlVE25 | Amp, *Leu2* marker, TEF promoter and XPR2 terminator  Amp, *Ura3* marker, TEF promoter and XPR2 terminator  pYLXP', D17-*P_TEF_-PaHPD-T_XPR2_*  pYLXP', D17-*P_TEF_-YlHPD-T_XPR2_*  pYLXP', E4-*P_TEF_-TrHPT-T_XPR2_*-*P_TEF_-AtVTE1-T_XPR2_*  pYLXP', E4-*P_TEF_-SyHPT-T_XPR2_*-*P_TEF_-AtVTE1-T_XPR2_*  pYLXP', A3-*P_TEF_-ARO1-T_XPR2_*  pYLXP', A3-*P_TEF_-ARO1-T_XPR2_*-*P_TEF_-ARO4-T_XPR2_*  pYLXP', A3-*P_TEF_-ARO1-T_XPR2_*-*P_TEF_-ARO7-T_XPR2_*  pYLXP', A3-*P_TEF_-ARO4-T_XPR2_*  pYLXP', A3-*P_TEF_-ARO4-T_XPR2_*-*P_TEF_-ARO7-T_XPR2_*  pYLXP', A3-*P_TEF_-ARO7-T_XPR2_*  pYLXP', F4-*P_TEF_-ARO1-T_XPR2_*  pYLXP', C7-*P_TEF_-ERG12-T_XPR2_*-*P_TEF_-YlGGPS-T_XPR2_*  pYLXP', E5-*P_TEF_-tHMG1-T_XPR2_*-*P_TEF_-IDI 1-T_XPR2_*  pYLXP', C1-*P_TEF_-ERG20-T_XPR2_*-*P_TEF_-SaGGPS-T_XPR2_*  pYLXP', C1-*P_TEF_-ERG20-T_XPR2_*-*P_TEF_-XdGGPS-T_XPR2_*  pYLXP', E2-*P_TEF_-SyHPT-*(TPTP)*-AtVTE1-T_XPR2_*  pYLXP', E2-*P_TEF_-SyHPT-*(TPTP)_2_*-AtVTE1-T_XPR2_*  pYLXP', E2-*P_TEF_-SyHPT-*(EAAAK)*-AtVTE1-T_XPR2_*  pYLXP', E2-*P_TEF_-SyHPT-*(EAAAK)_2_*-AtVTE1-T_XPR2_*  pYLXP', E2-*P_TEF_-SyHPT-*(GGGGS)*-AtVTE1-T_XPR2_*  pYLXP', E2-*P_TEF_-SyHPT-*(GGGGS)_2_*-AtVTE1-T_XPR2_*  pYLXP', E2-*P_TEF_-SyHPT-*(GSG)*-AtVTE1-T_XPR2_*  pYLXP', E2-*P_TEF_-SyHPT-*(GSG)_2_*-AtVTE1-T_XPR2_*  pYLXP', zeta-*P_TEF_-SyHPT^K77Y^-T_XPR2_*-*P_TEF_-AtVTE1-T_XPR2_*  pYLXP', zeta-*P_TEF_-SyHPT^K77Y^-T_XPR2_*-*P_TEF_-AtVTE1-T_XPR2_* | Our lab  Our lab  This study  This study  This study  This study  This study  This study  This study  This study  This study  This study  This study  This study  This study  This study  This study  This study  This study  This study  This study  This study  This study  This study  This study  This study  This study |

**Table S3. Primers used in this study**

| **Primers** | **Nucleotide sequence (5'-3')** |
| --- | --- |
| YLHPD-F  YLHPD-R  PaHPD-F  PaHPD-R  TrHPT-F  TrHPT-R  SyHPT-F  SyHPT-R  AtVTE1-F  AtVTE1-R  D17-UP-F  D17-DOWN-R  E4-UP-F  E4-DOWN-R  ARO1-F  ARO1-R  ARO4-F  ARO4-R  ARO7-F  ARO7-R  A3-UP-F  A3-DOWN-R  F4-UP-F  F4-DOWN-R  YlGGPS-F  YlGGPS-R  ERG12-F  ERG12-R  tHMG1-F  tHMG1-R  IDI 1-F  IDI 1-R  ERG20-F  ERG20-R  SaGGPS-F  SaGGPS-R  XdGGPS-F  XdGGPS-R  C7-UP-F  C7-DOWN-R  E5-UP-F  E5-DOWN-R  C1-UP-F  C1-DOWN-R  TPTP-F  TPTP-R  TPTP2-F  TPTP2-R  EAAAK-F  EAAAK-R  EAAAK2-F  EAAAK2-R  GGGGS-F  GGGGS-R  GGGGS2-F  GGGGS2-R  GSG-F  GSG-R  GSG2-F  GSG2-R | ttttgcagtactaaccgcagtcaccttccgtcgaagtcacc  catagcacgcgtgtagatacctaaaggttgcctcgcttggc  ttttgcagtactaaccgcagGCCGATATCTTTGAGAACCCCATGG  catagcacgcgtgtagatacTCAATCAGTAGACAGCACTCCTCTCC  ttttgcagtactaaccgcagCAGGCCACCACGGCC  catagcacgcgtgtagatactcaTTGGACAAAGGGGATAAGAAAGTACTCG  ttttgcagtactaaccgcagGCCACCATCCAAGCCTTCTG  catagcacgcgtgtagatacTTAGAAGATGGTGTTAGAGAAGTTGGGC  ctttttgcagtactaaccgcagGAGATCCGATCTCTGATTGTCTCAATGAAC  catagcacgcgtgtagatacTCATAGACCTGGTGGCTTGAAGAAAG  GATAGAGTCTCTTCAACTCGCTCAAAAAACAAC  GATTTAGGGTACCACCGGGTGAAG  cctctcagcatggggtttttttgt  gcacttgtacagtcgtctttacgaac  acgcaaagtgacaagctgtgcctagggacgacagagaccg  cggaggcttcactggacatgaactagatttgataacggacacggg  ttttgcagtactaaccgcagcccgctatgcacaacgcttc  catagcacgcgtgtagatacctaacctcgtcgagtcttgacgg  ttttgcagtactaaccgcaggacttcactaaagccgacaccg  catagcacgcgtgtagatacctactccaaccgccggagc  tggagtggaacttggacagtattaagaacg  agttcactttggacaccttctatggc  Ctgtacatacttgtatcttcattgccattgttgtatc  gacctcatgtcatgattgttggtgagg  ttttgcagtactaaccgcaggattataacagcgcggatttcaaggaga  catagcacgcgtgtagatactcactgcgcatcctcaaagtactt  TACACAGCACCACCTCAATCATGGACTACATCATTTCGGCGC  catagcacgcgtgtagatacCTAATGGGTCCAGGGACCGATG  ttttgcagtactaaccgcagaccCAGTCTGTGAAGGTGGTTG  catagcacgcgtgtagatacCTATGACCGTATGCAAATATTCGAACCg  ttttgcagtactaaccgcagACGACGTCTTACAGCGACAAAATC  catagcacgcgtgtagatacCTACTTGATCCACCGCCGAATCTC  ttttgcagtactaaccgcagtccaaggcgaaattcgaaagcG  catagcacgcgtgtagatacctacttctgtcgcttgtaaatcttggC  ttttgcagtactaaccgcagTCTTACTTCGACAACTACTTCAACGAGATC  catagcacgcgtgtagatacTTACTTTCGTCTTCGGATGGTGAACTC  ttttgcagtactaaccgcagGACTACGCCAACATCCTGACCG  catagcacgcgtgtagatacTTACAGGGGGATGTCGGCCAg  TCTCAGTGTGTCCAACCAAAGTGAG  TGCAACGTCCTACAATATATTATTTATTCGCGTATAC  gccaaccggttttactccttgC  gtgagtctcgttctcaacactattatcagatcatG  CAGTTGCGCATGAGACATCATCTAC  ACTATGGAGGGGCTGCGG  ATCTCCATCGGTGTCGGTGTTTAGAAGATGGTGTTAGAGAAGTTGGGC  TCTAAACACCGACACCGATGGAGATCCGATCTCTGATTGTCTCAATGAAC  CGGTGTCGGTGTCGGTGTCGGTGTTTAGAAGATGGTGTTAGAGAAGTTGGGC  CGACACCGACACCGACACCGATGGAGATCCGATCTCTGATTGTCTCAATGAAC  TCCATCTTGGCAGCGGCTTCTTAGAAGATGGTGTTAGAGAAGTTGGGC  AAGAAGCCGCTGCCAAGATGGAGATCCGATCTCTGATTGTCTCAATGAACC  GCAGCGGCTTCCTTGGCAGCGGCTTCTTAGAAGATGGTGTTAGAGAAGTTGGGCAG  GCTGCCAAGGAAGCCGCTGCCAAGATGGAGATCCGATCTCTGATTGTCTCAATGAACC  TCCATGCTACCGCCACCGCCTTAGAAGATGGTGTTAGAGAAGTTGGGCAG  AAGGCGGTGGCGGTAGCATGGAGATCCGATCTCTGATTGTCTCAATGAACC  CTACCGCCACCGCCGCTACCGCCACCGCCTTAGAAGATGGTGTTAGAGAAGTTGGGCAG  TGGCGGTAGCGGCGGTGGCGGTAGCATGGAGATCCGATCTCTGATTGTCTCAATGAACC  CGGATCTCCATGCCAGATCCTTAGAAGATGGTGTTAGAGAAGTTGGGCAG  TCTTCTAAGGATCTGGCATGGAGATCCGATCTCTGATTGTCTCAATGAACC  ATGCCAGATCCGCCAGATCCTTAGAAGATGGTGTTAGAGAAGTTGGGCAG  GGATCTGGCGGATCTGGCATGGAGATCCGATCTCTGATTGTCTCAATGAACC |

**Supplementary Figure**

**
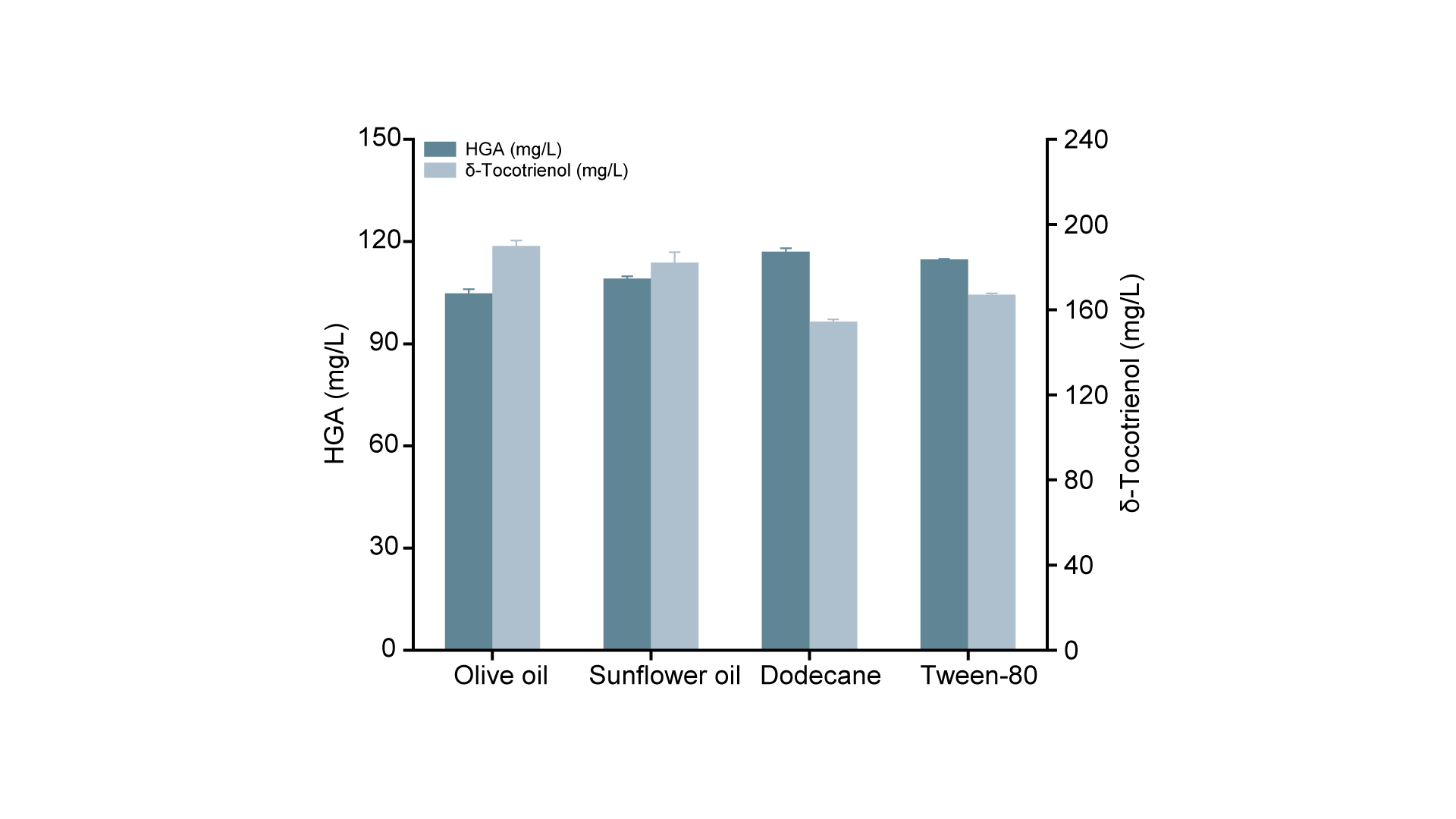
**

**Figure S1.** **Different *situ* extractants were added to the culture medium to promote the efflux of δ-tocotrienol. The strain VE-23 was used for fermentation in shake flasks.**

**
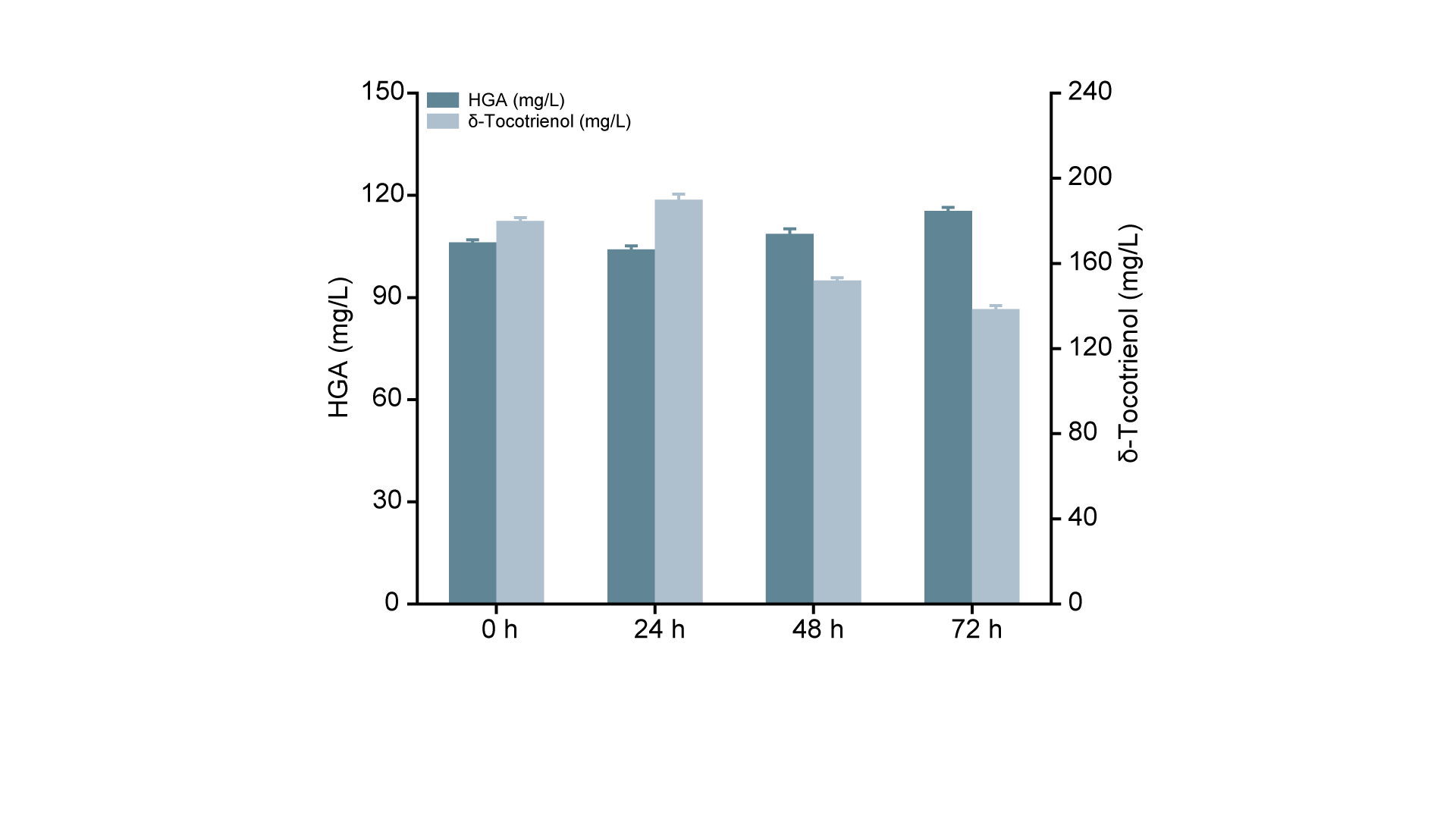
**

**Figure S2.** **Effect of the additional time of olive oil on the biosynthesis of δ-tocotrienol. The strain VE-23 was used for fermentation in shake flasks.**

**
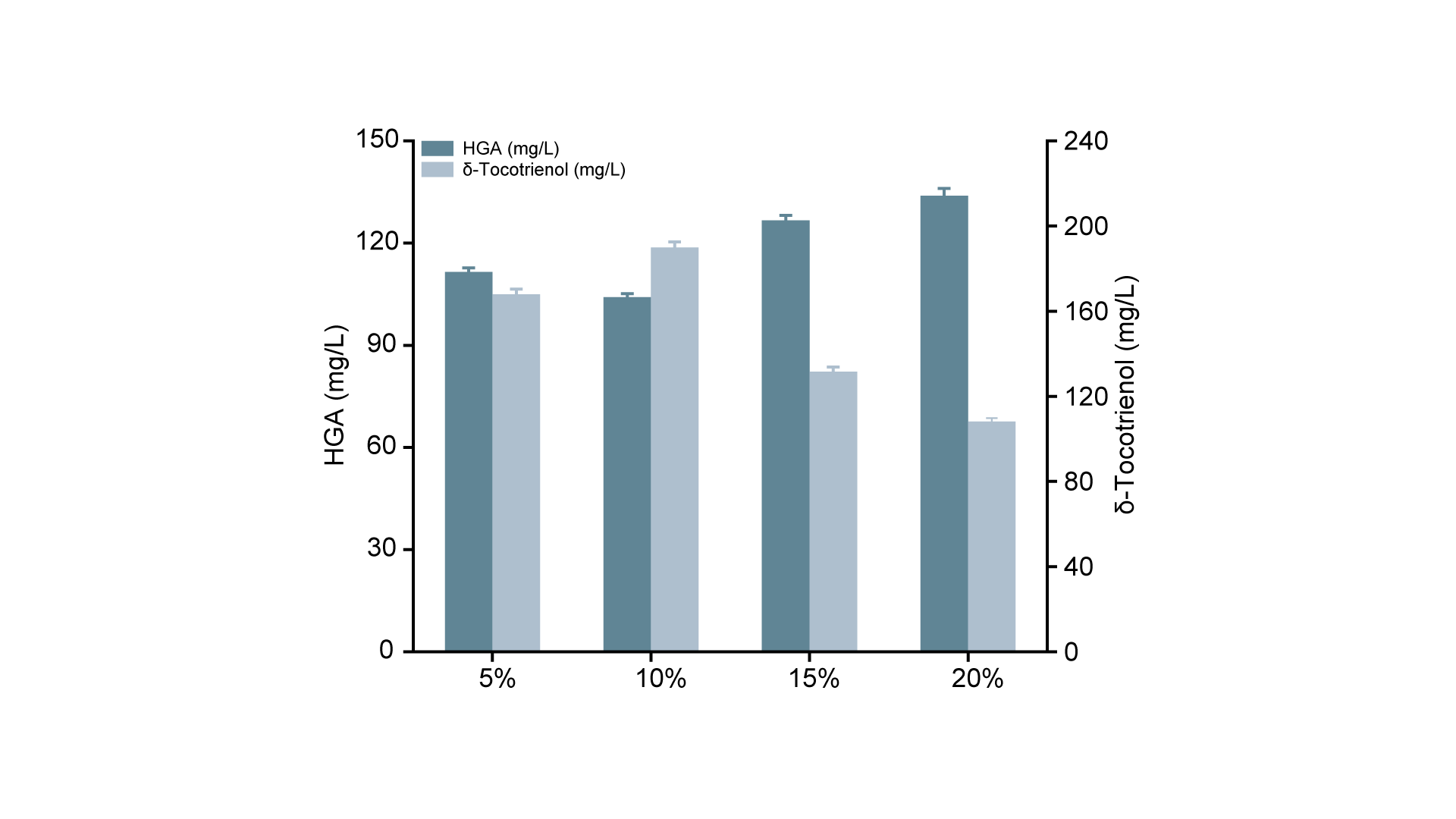
**

**Figure S3.** **Effect of the additional amount of olive oil on the biosynthesis of δ-tocotrienol. The strain VE-23 was used for fermentation in shake flasks.**


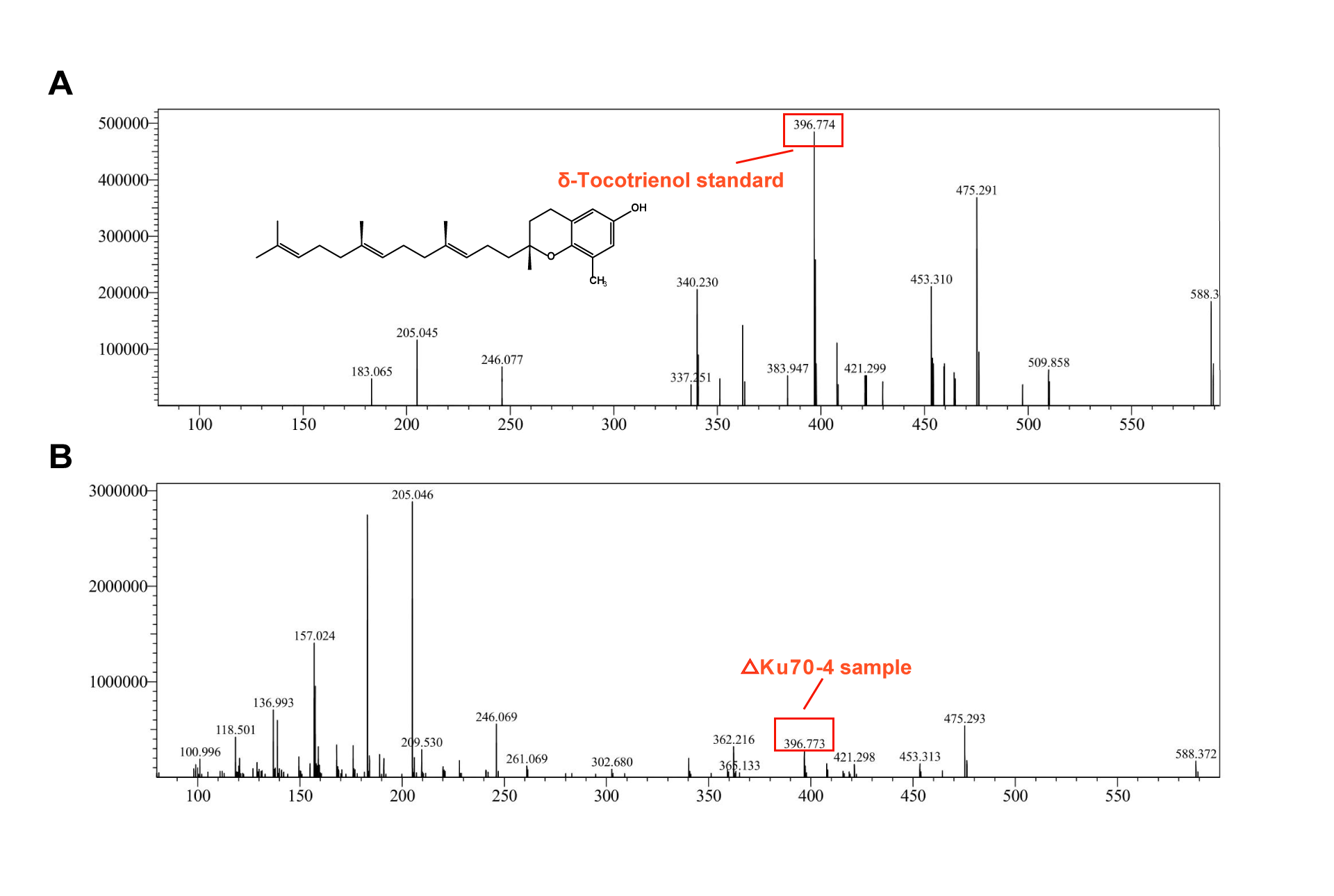


**Figure S4.** **LC-Q-TOF/MS Mass spectra of δ-Tocotrienol standard (A) and Δku70**

**-4 sample (B).**
